# HRCT features between lepidic-predominant type and other pathological subtypes in early-stage invasive pulmonary adenocarcinoma appearing as a ground-glass nodule

**DOI:** 10.1186/s12885-021-08821-5

**Published:** 2021-10-19

**Authors:** Pengju Zhang, Tianran Li, Xuemin Tao, Xin Jin, Shaohong Zhao

**Affiliations:** 1 Department of Radiology, Fourth Medical Center of PLA General Hospital, 51 Fucheng Road, Beijing, 100048 China; 2 Department of Radiology, First Medical Center of PLA General Hospital, 28 Fuxing Road, Beijing, 100853 China

**Keywords:** Lung neoplasms, Ground glass nodule, Tomography, X-ray computed, Pathology

## Abstract

**Background:**

Different pathological subtypes of invasive pulmonary adenocarcinoma (IPA) have different surgical methods and heterogeneous prognosis. It is essential to clarify IPA subtypes before operation and high-resolution computed tomography (HRCT) plays a very important role in this regard. We aimed to investigate the HRCT features of lepidic-predominant type and other pathological subtypes of early-stage (T1N0M0) IPA appearing as a ground-glass nodule (GGN).

**Methods:**

We performed a retrospective analysis on clinical data and HRCT features of 630 lesions in 589 patients with pathologically confirmed IPA (invasive foci > 5 mm) appearing as pure GGN (pGGN) and mixed GGN (mGGN) with consolidation-to-tumor ratio (CTR) ≤0.5 from January to December 2019. All GGNs were classified as lepidic-predominant adenocarcinoma (LPA) and nonlepidic-predominant adenocarcinoma (n-LPA) groups. Univariate analysis was performed to analyze the differences of clinical data and HRCT features between the LPA and n-LPA groups. Multivariate analysis was conducted to determine the variables to distinguish the LPA from n-LPA group independently. The diagnostic performance of different parameters was compared using receiver operating characteristic curves.

**Results:**

In total, 367 GGNs in the LPA group and 263 GGNs in the n-LPA group were identified. In the univariate analysis, the CTR, mean CT values, and mean diameters as well as mixed GGN, deep lobulation, spiculation, vascular change, bronchial change, and tumor–lung interface were smaller in the LPA group than in the n-LPA group (*P* <  0.05). Logistic regression model was reconstructed including the mean CT value, CTR, deep lobulation, spiculation, vascular change, and bronchial change (*P* <  0.05). Area under the curve of the logistic regression model for differentiating LPA and n-LPA was 0.840 (76.4% sensitivity, 78.7% specificity), which was significantly higher than that of the mean CT value or CTR.

**Conclusions:**

Deep lobulation, spiculation, vascular change, and bronchial change, CT value > − 472.5 HU and CTR > 27.4% may indicate nonlepidic predominant invasive pulmonary adenocarcinoma in GGNs.

## Background

With the development and popularization of high-resolution computed tomography (HRCT) lung cancer screening, the detection rate of ground-glass nodules (GGNs) has significantly increased [1, 2]. Numerous studies have confirmed that most long-term existed GGNs in the lung are mostly early lung adenocarcinoma or their precancerous lesions [3]. According to whether or not it contained a solid component, a GGN can be classified as pure GGN (pGGN) and mixed GGN (mGGN), a mGGN is divided into ground glass-predominant mGGN (0 < CTR ≤ 0.5) and solid-predominant mGGN (0.5 < CTR < 1) according to a consolidation-to-tumor ratio (CTR) on HRCT [4]. The 2015 World Health Organization (WHO) Classification of Lung Tumors [5] classified invasive pulmonary adenocarcinoma (IPA) into lepidic-predominant adenocarcinoma (LPA), acinar-predominant adenocarcinoma (APA), papillary-predominant adenocarcinoma (PPA), micropapillary-predominant adenocarcinoma (MPA), solid-predominant adenocarcinoma (SPA) according to the main growth patterns. Among them, LPA has the best prognosis, followed by the APA and PPA, whereas SPA and MPA have the worst [6, 7]. Different pathological subtypes of early-stage IPA may have different surgical methods, LPA may be treated with partial lobectomy (segmentectomy or wedge resection) and no lymph node dissection, whereas other subtypes can be treated with standard therapy for anatomical lobectomy and systemic lymph node dissection [7–9]. Preoperative puncture pathology and intraoperative freezing pathology are helpful for the diagnosis of pathological subtypes of lung adenocarcinoma, but both are limited by materials, and the risk of lung adenocarcinoma subtypes is underestimated [10]. HRCT can reflect the overall aggressiveness of nodules, and can help to improve the accuracy of pathological subtype diagnosis. Previous studies [11–13] have shown that HRCT features of GGN have high diagnostic value in differentiating early IPA from preinvasive lesions (PIL) and minimally invasive adenocarcinoma (MIA), but there are few studies on the correlation of HRCT features among subtypes of IPAs. Therefore, it is essential that HRCT features should be used to identify LPA from other subtypes in IPAs with GGN.

In this study, the HRCT features of IPAs appearing as pGGN and mGGN (CTR ≤ 0.5) were retrospectively analyzed to determine imaging differences between the LPA and other pathological subtypes to provide imaging help for clinical surgical decision-making.

## Methods

This study was approved by the Ethics Committee of Fourth Medical Center of Chinese PLA General Hospital (approval No. 2019YL002-HS001). All participants and/or their family members provided the informed consent.

### Participants

**Fig. 1:**
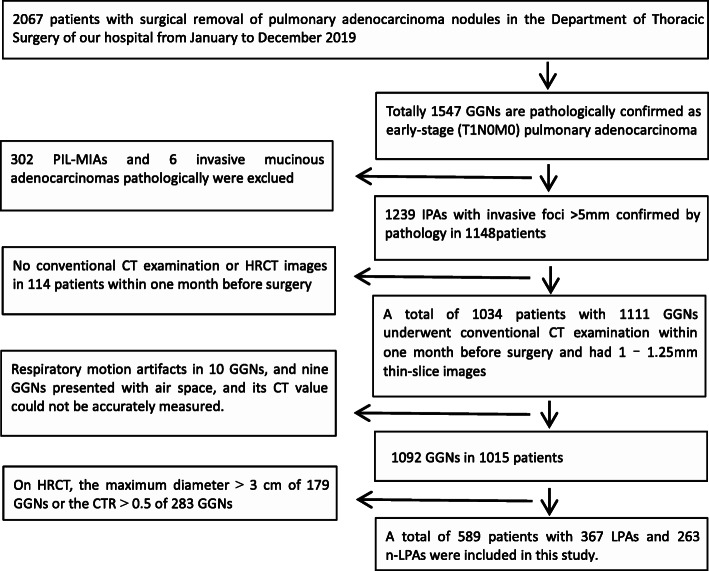
Flow chart of patient screening. HRCT, high-resolution computed tomography; IPA, invasive pulmonary adenocarcinoma; GGN, ground-glass nodule; PIL-MIA, preinvasive lesions-minimally invasive adenocarcinoma; CTR, consolidation-to-tumor ratio; LPA, lepidic-predominant adenocarcinoma; n-LPA, nonlepidic-predominant adenocarcinoma

The clinical and CT imaging data of patients having a confirmed diagnosis of IPA based on pathological results and undergoing resection at the Department of Thoracic Surgery of our hospital from January to December 2019 were collected. The inclusion criteria were as follows: (1) early-stage (T1N0M0) lung adenocarcinoma was confirmed by surgical pathology; (2) preoperative CT images appearing as GGN; (3) no chemoradiotherapy, no needle biopsy and no endoscopy were performed before the CT examination. The exclusion criteria were as follows: (1) PIL, MIA and variants of IPA; (2) no routine CT examination or 1 mm–1.25 mm thin-slice imaging performed within 1 month preoperatively; (3) heavy respiratory artifacts, or GGNs with air space type affecting the measurement of the CT value; (4) GGNs with a maximum diameter > 3 cm or CTR > 0.5 on CT. Finally, 589 patients (630 GGNs in total) were included for analysis (Fig. 1). Multiple GGNs in one patient were analyzed as independent lesions.

A total of 630 IPAs with invasive foci > 5 mm were divided into lepidic-predominant adenocarcinoma (LPA) and nonlepidic-predominant adenocarcinoma (n-LPA) groups. The subtypes of all IPAs in this study were recorded according to the pathology reports. The LPA group only included LPA subtype, whereas the n-LPA group included APA, PPA, MPA, and SPA subtypes. As a small number of micropapillary structures in IPA were strongly associated with poor prognosis [5], IPA with micropapillary structure was classified as n-LPA group in our study. One radiologist screened the participants and recorded the relevant clinical data.

### Inspection method

Brilliance iCT256 CT scanner (Philips Medical Systems, Netherland) or GE Optima 64 spiral CT scanner (GE Healthcare Technologies, Waukesha, WI) was used for conventional chest CT volume scanning. The scanning range was from the lung apex to the posterior costophrenic angle. The CT scan parameters were as follows: tube voltage, 120 KV; pitch, 0.993, 1.375; matrix, 1024 × 1024, 512 × 512; scanning slice thickness, 5 mm; reconstruction slice thickness, 1.25 mm or 1 mm; use of sharp and standard algorithms; lung window (window width, 1500 HU and window level, − 600 HU); and mediastinal window (window width, 400 HU and window level, 40 HU).

### Pathologic evaluation

The surgically resected specimens were fixed in 10% formalin, embedded in paraffin, sliced with a microtome, and stained with HE. All specimens were classified according to the criteria of the 2015 WHO Classification of Lung Tumors [5]. These tumors should be classified according to the main growth mode of the tumor and semiquantitatively evaluated the proportion of various growth patterns in 5% increments. The percentage of each growth pattern was recorded in the report. All included cases were reconfirmed by an experienced pulmonary pathologist.

### Analysis of clinical data and HRCT features

Clinical data were collected from the thoracic surgery database of our hospital, including patient’s age, sex, smoking history, and surgical records.

**Fig. 2:**
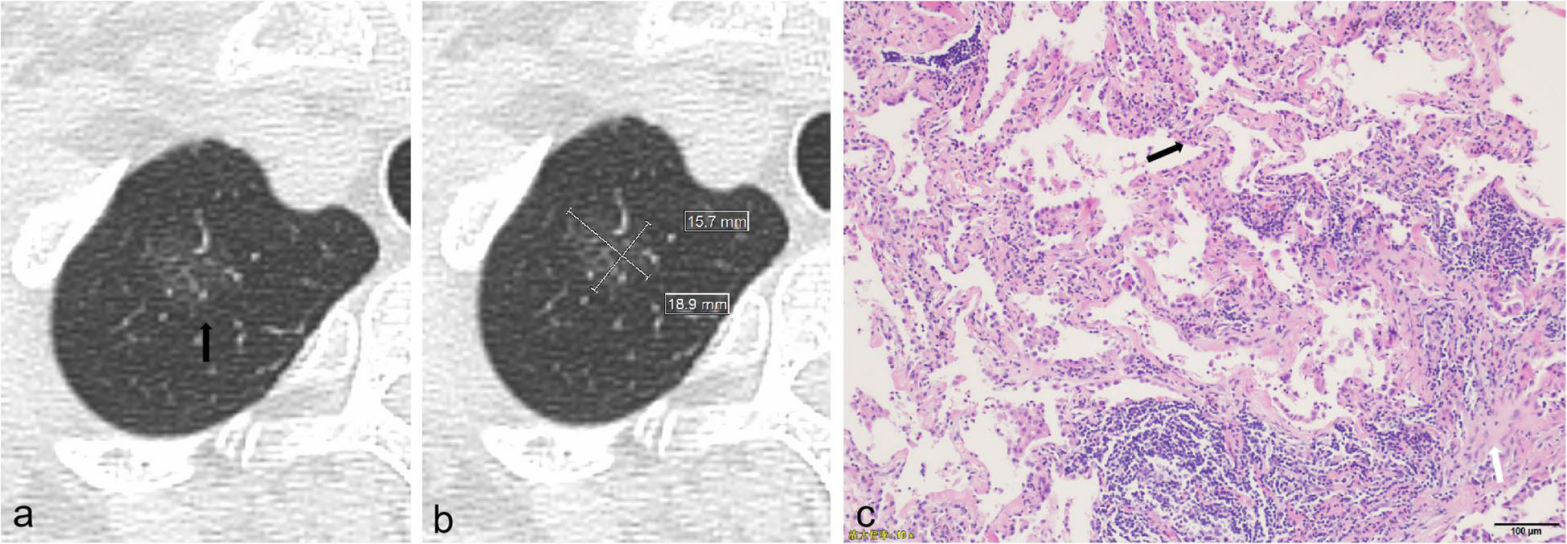
HRCT and pathological characteristics of a 72-year-old female patient with lepidic-predominant adenocarcinoma. (**a**) CT image showed a pGGN located in the right upper lobe with nonlobulated border and unclear tumor-lung interface (black arrow). (**b**) CT image showed that mean diameter was measured by calculating mean of longest diameter of nodule and its perpendicular diameter in same maximum view. (**c**) Photomicrograph of histopathologic specimen showed tumor cells lining preexisting alveolar structures with alveolar septum thickening (black arrow), foci of invasive components was more than 5 mm in thickness (white arrow). (Hematoxylin-eosin stain; original magnification, × 100)

The HRCT features, including continuous and categorical variables were browsed and analyzed on the Picture Archiving & Communication System. Several categorical variables included location, nodule type, deep lobulation, spiculation, vascular change, bronchial change, bubble-like lucency, pleural indentation, tumor–lung interface. The nodule type is pGGN and mGGN. Deep lobulation is characterized as a scallop-like lobulation on the nodule surface, and the maximum single lobulation chord distance/chord length is ≥0.4 (Fig. 2). Vascular change refers to the dilation, stiffness, distortion, and aggregation of blood vessels. Bronchial change refers to dilation, distortion, and truncation. The tumor–lung interface is a clear or fuzzy boundary between the tumor and adjacent normal lung tissue.

**Fig. 3:**
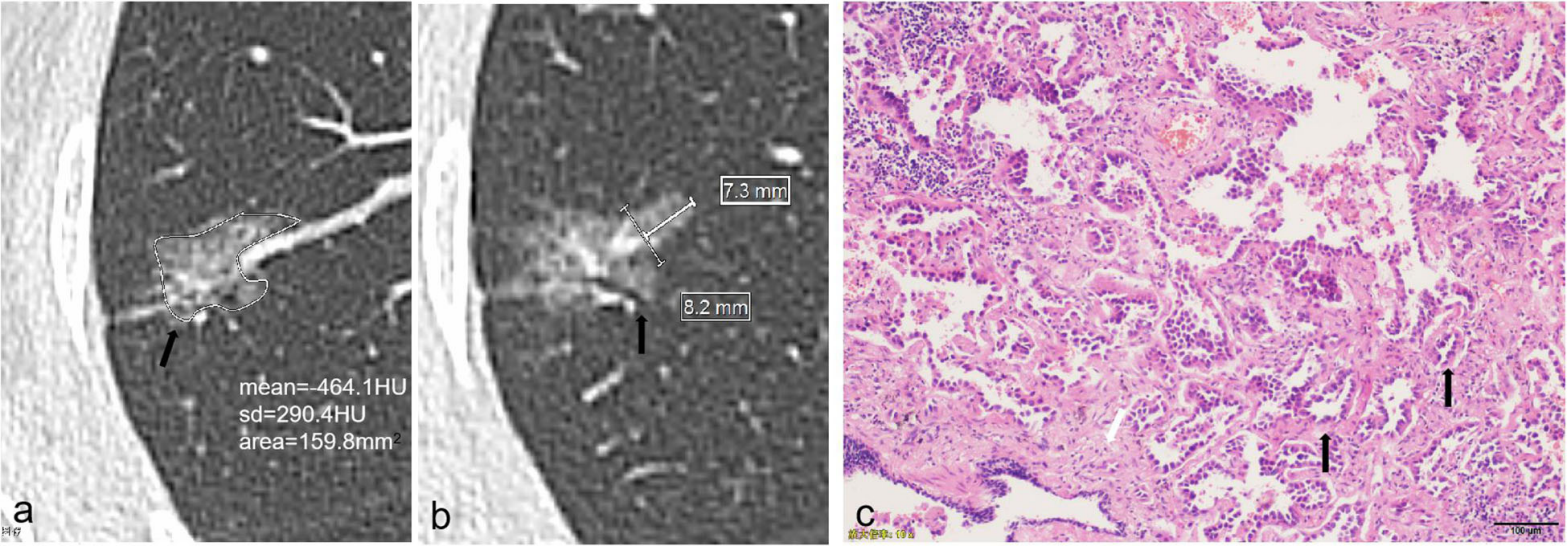
HRCT and pathological characteristics of a 51-year-old female patient with acinar-predominant adenocarcinoma. **a** CT image showed a mGGN in the right upper lobe with lobulated border, spiculated margin (black arrow), and pleural indentation, mean CT value was − 464.1HU, as measured using Freehand ROI (irregular white ring) as large as possible on axial view. **b** CT image showed that chordal distance (thick line)/chord length (thin line) of Deep lobular margin was 0.89 (7.3 mm/8.2 mm). vascular and bronchial changes (black arrow) were showed. **c** Photomicrograph of histopathologic specimen showed acinar pattern is consisting of round to oval shaped malignant glands (black arrow) invading fibrous stroma (white arrow). (Hematoxylin-eosin stain; original magnification, × 100)

**Fig. 4:**
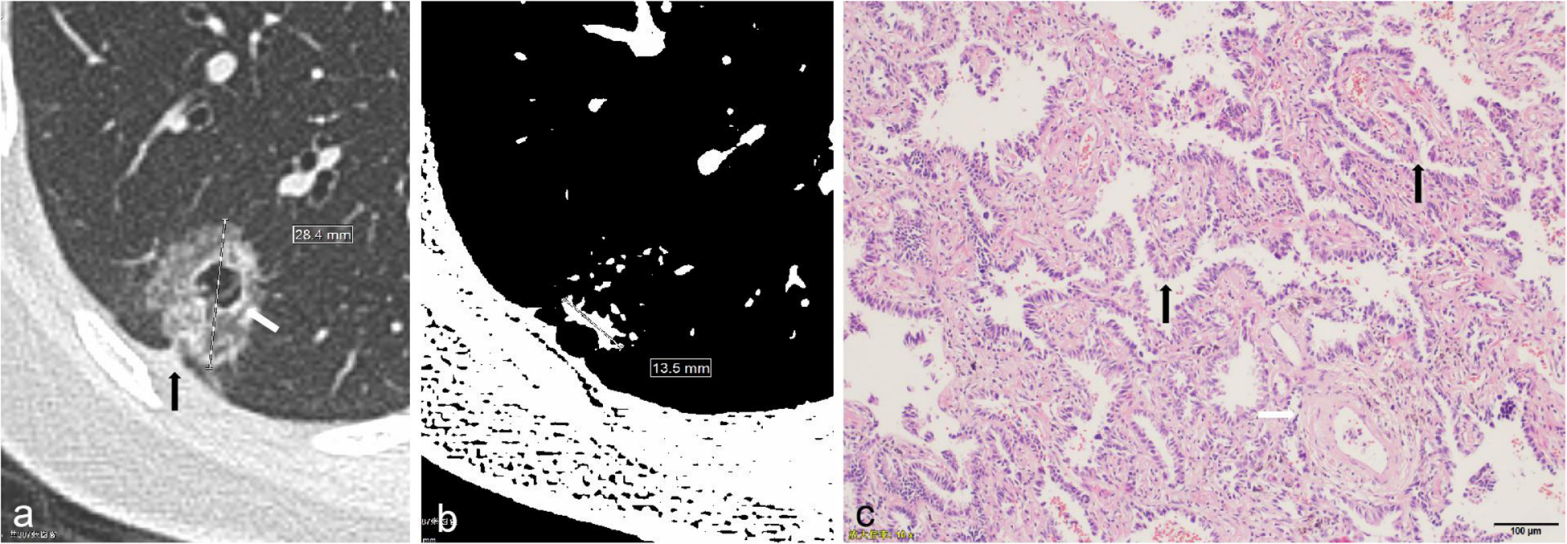
HRCT and pathological characteristics of a 55-year-old female patient with papillary-predominant adenocarcinoma. **a** CT image showed mGGN in the right lower lobe with spiculation, pleural indentation (black arrow) and bubble-like lucency (white arrow). The maximum diameter of GGN (Dmax) was measured. **b** CT image showed that the maximum diameter of solid component (Smax) was measured by adjusting window level and the window width (WL: -160HU; WD: 2HU). CTR = 47.5% (Smax/Dmax; 13.5 mm/28.4 mm). **c** Photomicrograph of histopathologic specimen showed a papillary pattern (black arrow) with cuboidal tumor cells growing along the surface of fibrovascular cores. Pulmonary vein was seen (white arrow). (Hematoxylin-eosin stain; original magnification, × 100)

Three continuous variables were record. The first variable was the mean diameter, which refers to the mean value of the longest diameter (D_max_) of the nodule measured at the maximum level of the lung window and the maximum diameter (D_per_) perpendicular to it (Fig. 3). The calculation method is as follows: (D_max_ + D_per_)/2. The second was CTR which is calculated by S_max_/D_max_ × 100%, S_max_ is the maximum diameter of solid component measured by adjusting window level and window width (WL, −160HU; WD, 2HU) [14] (Fig. 4). The last was the mean CT value which is measured by the Freehand region of interest (ROI) along the edge of the nodule on the maximum axis of GGN and upper and lower adjacent slices on the lung window, avoiding the bronchioles, blood vessels, and air-containing cysts as much as possible, and taking the mean value of three measurements as the mean CT value.

Two chest radiologists with > 15 years of working experience independently analyzed the images without knowing the clinical data and pathological diagnosis of the patients. Any inconsistent results were solved after discussion.

### Statistical analysis

When comparing the LPA and n-LPA groups, two independent sample *t* tests were used to assess continuous variables conforming to normal distribution, while Mann-Whitney U tests were used to compare non-normally distributed data. Continuous variables included age, mean diameter, mean CT value, and CTR. Categorical variables were analyzed using the Pearson *chi-square* test and Fisher’s exact test, including sex, smoking history, and HRCT features. The binary logistic regression analysis was performed on continuous and categorical variables with statistical significance in the univariate analysis. A simple logistic regression model was created using the backward elimination process. The receiver operating characteristic (ROC) analysis was performed using the logistic regression model and continuous variables with statistical significance in the multivariate analysis. The area under the curve (AUC) was used to evaluate the discrimination efficiency of the model in the LPA and n-LPA groups. The cutoff value was defined as the maximum value of the Youden’s index. All univariable and multivariate analyses were performed using IBM SPSS version 21.0 software. A *P*-value of < 0.05 was considered statistically significant.

## Results

The clinical findings of the patients were summarized in Table 1. Among 589 patients, 203 were men and 386 were women; the average age was 55 years (range, 23–78 years); 122 were smokers, 462 were nonsmokers, and five had an unknown smoking history. Of these patients, 260 patients underwent one-lobe lobectomy, 269 underwent single-wedge resection or segmental resection, one underwent two-lobe lobectomy (right upper lobe + middle lobe), 25 underwent one-lobe lobectomy combined with one-segmental or wedge resections, and 31 with two lesions and three with three lesions underwent multiple segmental or wedge resections. Of all GGNs, one GGN was detected in 553 patients, two GGNs in 32 patients, three in 3 patients and four in 1 patient.

**Table 1:** Clinical data analysis of GGN

Clinical information	Total number of patients (*n* = 589)	Number of GGNs (*n* = 630)		*P*
		LPA(367)	n-LPA(263)	
Age (year), mean ± standard deviation	55.03 ± 10.07	54.91 ± 9.82	55.26 ± 10.19	0.663
Sex				0.522
Male	203	129	86	
Female	386	238	177	
Smoking history (5 missing cases)				0.396
No	462	293	205	
Yes	122	70	58	

*GGN* Ground-glass nodule, *LPA* Lepidic-predominant adenocarcinoma, *n-LPA* Nonlepidic-predominant adenocarcinoma. *P* <  0.05 was considered statistically significant

Among 630 GGNs, 367 GGNs were in the LPA and 263 in the n-LPA groups including 221 APAs, 28 PPAs, three SPAs, and 11 IPAs with micropapillary structure. No significant differences were found in sex, age, and smoking history between the LPA and n-LPA groups (*P* = 0.522, 0.663, and 0.396, respectively).

### Analyzing HRCT features of GGNs

The mean diameter, mean CT value, and CTR were significantly smaller in the LPA group than in the n-LPA group (14.49 mm vs. 15.43 mm, *P* = 0.021; − 566.1 HU vs. − 449.3 HU, *P* <  0.001; 13.9% vs. 27.8%, *P* <  0.001). The location of GGN was as follows: 241 in the right upper lobe, 46 in the right middle lobe, 97 in the right lower lobe, 159 in the left upper lobe, and 87 in the left lower lobe. No significant difference was observed in the location, bubble-like lucency, and pleural indentation sign between the LPA and n-LPA groups (*P* = 0.477, 0.374, and 0.518, respectively). The mGGN, deep lobulation, spiculation, vascular change, bronchial change, and clear tumor–lung interface were more common in the n-LPA group than LPA group (*P* <  0.001, < 0.001, < 0.001, < 0.001, = 0003, and <  0.001, respectively) (Table 2).

**Table 2:** High-resolution computed tomography features of ground-glass nodules

Features	Classification	LPA (367)	n-LPA (263)	*P*
Mean diameter (mm), mean ± standard deviation		14.49 ± 5.22	15.43 ± 4.79	0.021
Mean CT value (HU), mean ± standard deviation		−566.1 ± 98.2	−449.3 ± 111.5	< 0.001
CTR(%), *M* (*Q1, Q3*)		13.9 (0, 27.3)	27.8 (7.7, 40)	< 0.001
Location	RUL	146	95	0.477
	RML	29	17	
	RLL	52	45	
	LUL	95	64	
	LLL	45	42	
Type	pGGN	262	149	< 0.001
	mGGN	105	114	
Deep lobulation	No	148	35	< 0.001
	Yes	219	228	
Spiculation	No	280	101	< 0.001
	Yes	87	162	
Vascular change	No	206	53	< 0.001
	Yes	161	210	
Bronchial change	No	92	40	0.003
	Yes	275	223	
Bubble-like lucency	No	314	218	0.374
	Yes	53	45	
Pleural indentation sign	No	178	120	0.518
	Yes	189	143	
Tumor–lung interface	Unclear	122	46	< 0.001
	Clear	245	217	

*LPA* Lepidic-predominant adenocarcinoma, *n-LPA* Nonlepidic-predominant adenocarcinoma, *CTR* Consolidation-to-tumor ratio, *pGGN* Pure ground-glass nodule, *mGGN* Mixed ground-glass nodule, *RUL* Right upper lobe, *RML* Right middle lobe, *RLL* Right lower lobe, *LUL* Left upper lobe, *LLL* Left lower lobe. *P* <  0.05 was considered statistically significant

### Logistic regression analysis and ROC analysis of GGNs

Binary logistic regression analysis was performed on HRCT features with statistical significance in univariate analysis, including mean diameter, mean CT value, CTR, deep lobulation, spiculation, vascular change, bronchial change, and tumor–lung interface. As nodule type was strongly associated with CTR, nodule type was excluded from logistic regression analysis. Binary logistic regression analysis showed that high CT value [*P* <  0.001; odds ratio (OR) = 1.009], deep lobulation (*P* <  0.001; OR = 3.250), spiculation (*P* = 0.002; OR = 1.949), vascular change *P* <  0.001; OR = 2.571), and bronchial change (*P* = 0.036; OR = 1.745) were independent predictors of increasing the risk of the n-LPA group, whereas small CTR (*P* = 0.007; 0.982) was a factor that reduced the risk of the n-LPA group (Table 3).

**Table 3:** Ability to distinguish IPA between the LPA group and n-LPA group using the backward elimination process of binary logistic regression

Feature	B	SE	*P* value	OR	95% CI
Mean CT value	0.009	0.001	< 0.001	1.009	1.007, 1.011
CTR	−0.018	0.007	0.007	0.982	0.969, 0.995
Deep lobulation	1.179	0.257	< 0.001	3.250	1.962, 5.382
Spiculation	0.667	0.216	0.002	1.949	1.275, 2.977
Vascular change	0.944	0.231	< 0.001	2.571	1.636, 4.041
Bronchial change	0.557	0.265	0.036	1.745	1.037, 2.935
Constant	2.346	0.660	< 0.001	10.441	

*LPA* Lepidic-predominant adenocarcinoma, *n-LPA* Nonlepidic-predominant adenocarcinoma, *CTR* Consolidation-to-tumor ratio, *SE* Standard error, *OR* Odds ratio (OR > 1 indicates the risk factor of n-LPA; OR < 1 indicates the protective factors of n-LPA). *P* < 0.05 was considered statistically significant

**Fig. 5:**
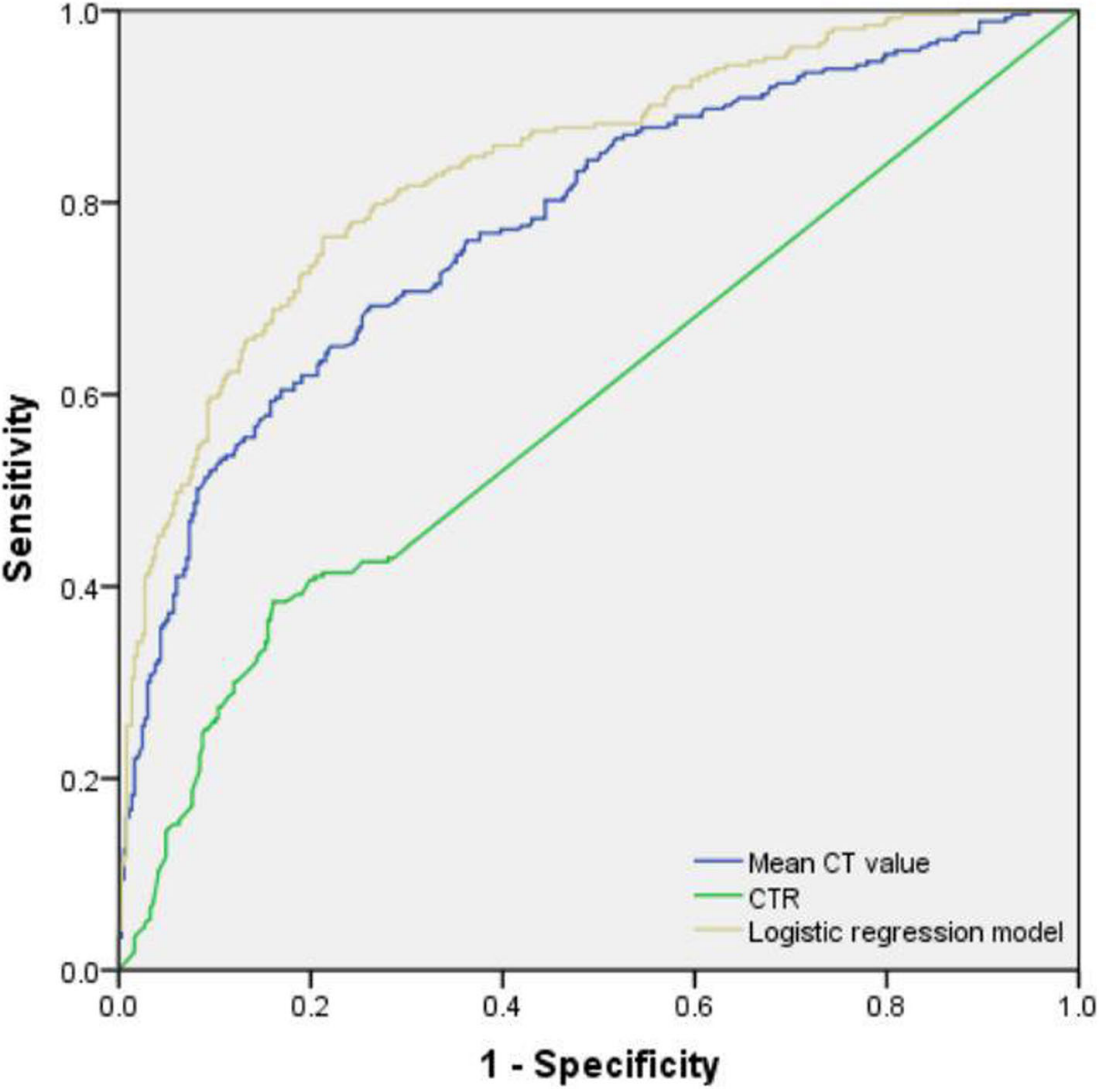
Ability of the mean CT value, CTR, and logistic regression model to distinguish LPA from n-LPA. Logistic regression model includes mean CT value, CTR, deep lobulation, spiculation, vascular change, and bronchial change. The AUC value (AUC = 0.840) was significantly higher than the mean CT value (AUC = 0.781) or CTR (AUC = 0.593), and the diagonal line represents the reference line

ROC analysis was performed on the mean CT value, CTR, and logistic regression model with significant differences in multivariate analysis, which showed that the AUC was 0.781 [95% confidence interval (CI) 0.744–0.818], 0.593 (95% CI: 0.547–0.639), and 0.840 (95% CI: 0.808–0.871), respectively. The optimal cutoff values of the mean CT and CTR were − 472.5 HU (sensitivity, 60.5%; specificity, 83.1%) and 27.4% (sensitivity, 38.4%; specificity, 83.9%), respectively. Whereas the sensitivity and specificity of logistic regression model were 76.4 and 78.7%, respectively (Table 4). Logistic regression model conbined with mean CT value, CTR, and morphological HRCT features had significantly higher predictive efficiency than that using only mean CT value or CTR (*P* <  0.001) (Fig. 5).

**Table 4:** Comparison of the mean CT value, CTR, and ROC curve of the logistic regression model to distinguish IPA between the n-LPA and LPA groups

Factor	AUC value (95% confidence interval)	Cutoff value	Sensitivity (%)	Specificity (%)	*P*
Mean CT value	0.781 (0.744–0.818)	−472.5 HU	60.5%	83.1%	< 0.001
CTR	0.593 (0.547–0.639)	27.4%	38.4%	83.9%	< 0.001
Logistic model	0.840 (0.808–0.871)	3.958	76.4%	78.7%	< 0.001

Cutoff value: The cutoff value of ROC curve of logistic regression model is the factor value or predictive probability value of the model and the maximum value of Youden’s index. *LPA* Lepidic-predominant adenocarcinoma, *n-LPA* Nonlepidic-predominant adenocarcinoma, *CTR* Consolidation-to-tumor ratio, *AUC* Area under the curve. *P* < 0.05 was considered statistically significant

## Discussion

To our knowledge, LPA has the best prognosis without lymph node dissection or only lymph node sampling, whereas n-LPA requires systematic lymph node dissection [8], so we performed a retrospective analysis of 630 GGNs that were identified pathologically after resection as early-stage IPA with invasive foci more than 5 mm. Previous studies [11–13] has confirmed that HRCT features had good diagnostic value in differentiating IPA from PIL-MIA, therefore, we excluded PIL and MIA cases and focused on differences in the HRCT features of IPA subtypes. Our previous studies also showed that even with pGGN, 39% were various subtypes of IPAs [15]. In this cohort, we included pGGNs and mGGNs with CTR ≤ 0.5, and pGGNs accounted for 65.2%. The number of LPA was greater than that of n-LPA (367/263), and the proportion of pGGNs in the LPA group was significantly higher than that in the n-LPA group (*P* <  0.001). This was consistent with the previous report that the ground-glass opacity on HRCT was positively correlated with the lepidic growth pattern of the tumor pathologically [7]. In GGNs, the emergence of various IPA subtypes may be related to the following aspects, APA and PPA appeared as GGN for air-filled sparse hypertrophic areas [13]; This study included three SPAs, which were composed of a mixture of solid-predominant growth pattern and other growth patterns, of which lepidic growth patterns accounts for 40%, 30%, 30%, and their diameters were all less than 10 mm; MPA is usually manifested as solid nodules, and the micropapillary type in our study was other subtypes containing a small number of micropapillary structures, so it can be manifested as GGN; And beyond that, This discrepancy between radiological and pathological findings could be explained by a partial volume effect, detection of small nonaerated components may be difficult because of inadequate spatial resolution [13]. Although, our all CT images were acquired by using HRCT at 1 mm or 1.25mm slice thickness, a much higher resolution may be needed to detect small nonaerated invasion.

This was the larger study of GGNs for the purpose of differentiating LPA from n-LPA group. We observed that the ratio of IPA was significantly higher in women (65.5%) and no smokers (79.1%). The Fleischner Society Guidelines [2] also referred to the incidence of adenocarcinoma in nonsmokers was increasing, with female nonsmokers being affected significantly more often than male nonsmokers. However, the relationship to nodule type was not reported. In our study, there were no significant differences between LPA and n-LPA groups in both sex and smoking history.

In univariate and multivariate analysis showed that deep lobulation, spiculation, vascular change and bronchial change were more common in the n-LPA group than in the LPA group. (*P* <  0.05). Several studies have examined marginal lobulation and spiculation related to “tumor development or invasion” in adenocarcinoma [2, 5, 12]. Zhang et al. [16] showed bronchial change was a predictor of invasiveness. Gao et al. [17] found III and IV vascular changes were more prone to invasive behavior, where type III vessels within lesions are tortuous or rigid without an increase in amount and type IV vessels are more complex vascular changes than the other types, such as irregular expansion and convergence of multiple blood vessels. Liang et al. [18] also demonstrated that the number of blood vessels entering GGN (vascular aggregation) was a risk factor for predicting invasiveness. These morphologic features were associated with active fibroblast proliferation in adenocarcinoma and caused by the contraction of fibrous tissues [19]. Noguchi et al. [20] suggested that active fibroblast proliferation in adenocarcinoma was related to the invasive growth of tumors.

Previous studies [13, 14] comparing IPA with PIL-MIA had shown that the CT value of GGN was confirmed to be associated with invasiveness. For example, Zhou et al. [11] showed that the lesion with CT values in pGGN and mGGN greater than − 583.6HU and − 571.6HU, respectively, was more likely to be IPA. Lee et al. [12] showed that GGNs with CT values of > − 472HU were more likely to be IPAs. This result was similar to the mean CT value (> − 472.5HU) we predicted for the n-LPA, theoretically, however, the mean CT value threshold of IPA subtypes should be greater than that of IPA and MIA-PIL. This may be due to the fact that we used the Freehand ROI but not Circle ROI to draw along the edge of the GGN as large as possible on the maximum transverse axis on the lung window, but Lee used the circular ROI outline causing the loss of the edge part with large gas content for irregular nodules, thus raising the CT value. However, considering the influence of CT scanning parameters on the CT value of GGN, it was limited to make a diagnosis only by CT value. In this study, CTR > 27.4% was more likely to be n-LPA with poor prognosis, which was consistent with the conclusion by Tsutani et al. [21] that showed GGN with CTR > 25% had a high postoperative recurrence rate. Ko et al. [22] studied 138 cases of stage I lung adenocarcinoma with GGN, and the CTR of LPA and n-LPA were 14.5 and 35.4% (*P* = 0.002), which were higher than the CTR of LPA (13.9%) and n-LPA (27.8%) in this study. Considerations were first related to the exclusion of mGGNs with CTR > 0.5, which reduced the proportion of solid predominant GGNs in the n-LPA group, however, LPA was rare in mGGNs with CTR > 0.5, so it was less affected in LPA, and secondly, the measurement methods of the two studies were inconsistent.

In univariate analysis, there were statistically significant differences in the mean diameter and tumor–lung interface (*P* = 0.021, < 0.001), but not in multivariate analysis. Some studies [12, 13] showed the size of the nodule was related to the malignancy of the tumor, however the pathological subtypes of IPA were determined by the proportion of invasive component which was more common in the central region of the GGN [19], while the size of the nodule was determined by the peripheral lepidic growth pattern progressing slowly. In this study, the mean diameter of GGN in the LPA group and the n-LPA group overlapped greatly (14.49 ± 5.22 mm:15.43 ± 4.79 mm), so it could not be used as an independent predictor. Hwang et al. [23] also showed that in patients with early pulmonary adenocarcinoma measuring < 3 cm, disease-free survival was remarkably correlated with the size of the solid part of the tumor, but not with the whole tumor. A fuzzy tumor–lung interface was more likely to occur in the LPA group than in the n-LPA group. The lepidic growth pattern part of LPA was more common than that of n-LPA. the lepidic growth pattern was that the tumor cells growing along thickened alveolar walls and air filled in the alveolar cavity [12], when the air content of the peripheral part was close to that of adjacent normal lung tissue and the CT spatial resolution was limited, the tumor–lung interface of GGN on CT was fuzzy. However, the frequency of clear tumor–lung interface was higher in both groups and was not statistically significant in multivariate analysis. No significant differences were observed in pleural indentation sign and bubble-like lucency. Masahiro et al. [24] found that the incidence of pleural indentation increased with increased tumor invasiveness, the volume ratio of solid components in GGN was > 63% and that the incidence of pleural indentation sign increased. Nevertheless, we investigated GGNs with CTR of < 50% and did not consider the distance between the nodules and pleura. The incidence of bubble-like lucency in both groups was low, which was related to the observation of multi-directional reconstruction images, excluding the air space connected with the bronchus.

Our study also found that AUC values of the logistic regression model, the mean CT values and CTR were 0.840 (sensitivity, 76.4%, specificity, 78.7%), 0.781 (sensitivity, 60.5%, specificity, 83.1%), and 0.593 (sensitivity, 38.4%, specificity, 83.9%), respectively. The logistic regression model, which obtained by combining deep lobulation, spiculation, vascular change, and bronchial change, the mean CT values and CTR, can improve the sensitivity more than using mean CT value or CTR alone in distinguishing LPA from n-LPA. Differentiating LPA and n-LPA was very important for the preoperative planning of surgical procedures and simultaneously as a reference value for GGN management. For GGN in patients with deep lobulation and vascular and bronchial changes, the follow-up should be terminated and surgical treatment should be considered.

This study has certain limitations. First, this was a retrospective analysis, mainly based on pathological diagnosis. The assessment of IPA subtypes may be inconsistent, especially when multiple subtype components coexisted. Second, this study only focused on lepidic and nonlepidic types of IPA and did not further analyze imaging differences among other pulmonary adenocarcinomas.

## Conclusions

The HRCT features, including deep lobulation, spiculation, vascular change, and bronchial change, may indicate nonlepidic IPA in GGNs, and the diagnosis of nonlepidic IPA is supported when the mean CT value is > − 472.5 HU or CTR is > 27.4%. Simultaneously, the combined application of mean CT value, CTR, and HRCT features can effectively improve the diagnosis of nonlepidic.

## Data Availability

The dataset used and/or analyzed during the current study are available from corresponding author on reasonable request.

## Abbreviations

PLA: People’s Liberation Army
HRCT: High-resolution computed tomography
IPA: Invasive pulmonary adenocarcinoma
GGN: Ground-glass nodule
pGGN: Pure GGN
mGGN: Mixed GGN
CTR: Consolidation-to-tumor ratio
WHO: World Health Organization
PIL: Preinvasive lesions
MIA: Minimally invasive adenocarcinoma
LPA: Lepidic-predominant adenocarcinoma
APA: Acinar-predominant adenocarcinoma
PPA: Papillary-predominant adenocarcinoma
MPA: Micropapillary-predominant adenocarcinoma
SPA: Solid-predominant adenocarcinoma
n-LPA: Nonlepidic-predominant adenocarcinoma
iCT: Information and Communication technology
GE: General Electric Company
IBM: International Business Machine
WI: Wisconsin
HU: Hounsfield unit
KV: Kilovolt
WL: window level
WD: Window width
HE: Hematoxylin-eosin
ROC: Receiver operating characteristic
AUC: Area under the curve
OR: Odds ratio
CI: Confidence interval
SE: Standard error
M: Median
Q1: Quartile1
Q3: Quartile3
vs: Versus
RUL: Right upper lobe
RML: Right middle lobe
RLL: Right lower lobe
LUL: Left upper lobe
LLL: Left lower lobe
R&D: Research and development
